# Long-Term Effects With Potential Clinical Importance of Botulinum Toxin Type-A on Mechanics of Muscles Exposed

**DOI:** 10.3389/fbioe.2020.00738

**Published:** 2020-06-30

**Authors:** Cemre S. Kaya, Evrim O. Yılmaz, Zeynep D. Akdeniz-Doğan, Can A. Yucesoy

**Affiliations:** ^1^Institute of Biomedical Engineering, Boğaziçi University, Istanbul, Turkey; ^2^Department of Plastic Reconstructive and Aesthetic Surgery, Marmara University, Istanbul, Turkey

**Keywords:** botulinum toxin type A, muscle mechanical function, active force, passive force, collagen, animal model

## Abstract

Botulinum toxin type-A (BTX-A) is widely used for spasticity management and mechanically aims at reducing passive resistance at the joint and widening joint range of movement. However, recent experiments on acute BTX-A effects showed that the injected rat tibialis anterior (TA) muscle’s passive forces increased, and the length range of active force exertion (*l*_range_) did not change. Additionally, BTX-A was shown to spread into non-injected muscles in the compartment and affect their mechanics. Whether those effects persist in the long term is highly important, but unknown. The aim was to test the following hypotheses with experiments conducted in the anterior crural compartment of the rat: In the long term, BTX-A (1) maintains *l*_range_, (2) increases passive forces of the injected TA muscle, and (3) spreads into non-injected extensor digitorum longus (EDL) and the extensor hallucis longus (EHL) muscles, also affecting their active and passive forces. Male Wistar rats were divided into two groups: BTX-A and Control (0.1 units of BTX-A or only saline was injected into the TA). Isometric forces of the muscles were measured simultaneously 1-month post-injection. The targeted TA was lengthened, whereas the non-targeted EDL and EHL were kept at constant length. Hydroxyproline analysis was done to quantify changes in the collagen content of studied muscles. Two-way ANOVA test (for muscle forces, factors: TA length and animal group) and unpaired *t* or Mann-Whitney *U* test (for *l*_range_ and collagen content, where appropriate) were used for statistical analyses (*P* < 0.05). BTX-A caused significant effects. TA: active forces decreased (maximally by 75.2% at short and minimally by 48.3%, at long muscle lengths), *l*_range_ decreased (by 22.9%), passive forces increased (by 12.3%), and collagen content increased (approximately threefold). EDL and EHL: active forces decreased (up to 66.8%), passive force increased (minimally by 62.5%), and collagen content increased (approximately twofold). Therefore, hypothesis 1 was rejected and 2 and 3 were confirmed indicating that previously reported acute BTX-A effects persist and advance in the long term. A narrower *l*_range_ and an elevated passive resistance of the targeted muscle are unintended mechanical effects, whereas spread of BTX-A into other compartmental muscles indicates the presence of uncontrolled mechanical effects.

## Introduction

A widely used technique for management of spasticity arising from a wide range of conditions such as cerebral palsy (CP) (Graham et al., 2000; Criswell et al., 2006; Lukban et al., 2009), spinal cord injury (Adams and Hicks, 2005; Marciniak et al., 2008), multiple sclerosis (Hyman et al., 2000; Van Der Walt et al., 2012) and stroke (Bakheit et al., 2000; Bhakta et al., 2000) is injection of botulinum toxin type-A (BTX-A). The toxin temporarily paralyzes muscles by inhibiting the discharge of the acetylcholine containing vesicles into the synaptic cleft and hence transmission of nerve impulses to the muscle fibers at the neuromuscular junction (Blasi et al., 1993; Brin, 1997; Hammond et al., 2015). A consequence is decreased muscle tone (Grazko et al., 1995; Whelchel et al., 2004) which mechanically implies a limited muscular force production capacity. BTX-A treatment aims at improving joint function (Love et al., 2001) by reducing the passive resistance of the muscle in the joint (Sheean, 2001) and increasing the joint range of motion (Koman et al., 2000).

Understanding BTX-A effects on muscular mechanics is of central importance because muscle exposed continues to serve as the motor for movement, but this understanding remains limited. Length-dependent reductions in knee extension torque (Longino et al., 2005) and muscle force (Yucesoy et al., 2012) shown in previous animal experiments indicate complex effects of BTX-A on joint mechanics. Additionally, experiments in the rat anterior crural compartment have shown acutely that BTX-A does not improve the muscle’s length range of force exertion (*l*_range_) and elevates passive forces of the injected tibialis anterior (TA) muscle (Yucesoy et al., 2012). In addition, spread of BTX-A through muscle fascia was reported (Shaari et al., 1991). Such spread has been reported to reduce forces (Yaraskavitch et al., 2008; Frasson et al., 2012) and cause changes in length-force characteristics of also the non-injected muscles (Yucesoy et al., 2012; Ates and Yucesoy, 2014, 2018; Yucesoy and Ates, 2018). Remarkably, those changes in the short term include effects contradicting treatment aims (i.e., decreased *l*_range_, increased passive forces and elevated intramuscular collagen content) (Ates and Yucesoy, 2014). Finite element analyses of that indicated that they do ascribed to a continuing elevated stiffness the exposed muscles’ extracellular matrix (Turkoglu and Yucesoy, 2016), testing of which deserves major attention.

Whether the short-term effects persist in the long term is unknown, but very important. Therefore, in a rat model, we aimed at testing the following hypotheses. In the long term, BTX-A (1) maintains *l*_range_, (2) increases the passive forces of the injected TA muscle, and (3) spreads into non-injected muscles also affecting their active and passive forces.

## Materials and Methods

### Assessment of the Effects of BTX-A on Muscular Mechanics

Surgical and experimental procedures were approved by the Committee on the Ethics of Animal Experimentation at Boğaziçi University. Male Wistar rats were divided into two groups: control (*n* = 7; mean ± SD: body mass 386.3 ± 36.5 g and 406.9 ± 16.8 g for the times of injection and experiment, respectively) and BTX-A (*n* = 7; body mass 394.7 ± 29.0 g and 404.3 ± 34.3 g for the times of injection and experiment, respectively).

Using intraperitoneal ketamine (1 mg/kg), a mild sedation was imposed. Subsequently, a region was shaved, bound within an approximately 15 mm radius from the center of the patella, where a marker was placed. Brining the ankle to maximal plantarflexion and the knee to approximated 90° angle, the TA muscle was located by palpation. At a point 10 mm distal along the tibia, a second marker was placed. A line segment was drawn between the two markers and the injection location over the TA muscle was determined as a point 5 mm lateral to the second marker. At this location, the depth of the TA and the thickness of the skin approximated 5–5.5 and 0.7–1 mm, respectively. All injections were made exclusively into the TA, to a depth of 3 mm, therefore into the superficial half of the muscle.

A 100 U vial of vacuum-dried, botulinum type A neurotoxin complex (BTX-A) (BOTOX; Allergan Pharmaceuticals, Westport, Ireland) was reconstituted with 0.9% saline solution. For the BTX-A group, the animals received a one-time intramuscular BTX-A injection. The total dose was 0.1 U and the injected volume was 20 μl. For the control group, the animals received a one-time intramuscular injection of the same volume of 0.9% saline solution exclusively. All injections were performed 1-month prior to testing. The animals were kept in standard cages separately. The animal care room was thermally regulated and maintained a 12 h dark-light cycle. The animals were free to do their normal activity until the day of the experiment.

#### Surgical Procedures

The animals were anesthetized using an intraperitoneally injected urethane solution (1.2 ml of 12.5% urethane solution per 100 g of body mass). Additional doses were given if necessary (maximally 0.5 ml). Immediately following the experiments, the animals were euthanized by using an overdose of urethane solution.

To prevent hypothermia, the animals were kept on a heated pad (Homoeothermic Blanket Control Unit; Harvard Apparatus, Holliston, MA, United States) during surgery and data collection. The control of the body temperature at 37°C was obtained by adjusting the temperature of the heated pad utilizing a feedback system integrated with a rectal thermometer.

During surgery, the skin and the biceps femoris muscle of the left hindlimb were removed. After the anterior crural compartment was exposed, only a limited distal fasciotomy was performed to remove the retinaculae (i.e., the transverse crural ligament and the crural cruciate ligament). Consequently, the connective tissues of the muscle bellies within the compartment [i.e., the TA, extensor digitorum longus (EDL) and extensor hallucis longus (EHL) muscles] were left intact.

A reference position was selected as the combination of knee joint angle of 120°; and ankle angle of 100°. This position matches with a combination of knee and ankle positions that the rat attains *in vivo*, during the stance phase of gait (Gruner et al., 1980). Maintaining the reference position, the following were done: using silk thread, the four distal tendons of the EDL muscle were tied together. On the distal tendons of the EDL, TA, and EHL muscles, as well as on a fixed location on the lower leg matching markers were placed. Afterward, the distal EDL tendon complex as well as the TA and the EHL tendons were cut as distally as possible.

The femoral compartment was exposed for two purposes: (1) to reach the proximal tendon of the EDL and (2) to expose the sciatic nerve. Subsequently, keeping a small piece of the lateral femoral condyle still attached, the tendon was cut from the femur. The sciatic nerve was dissected free of other tissues. Once all nerve branches to the muscles of the femoral compartment were severed, the sciatic nerve was cut as proximally as possible.

The proximal tendon of the EDL, the tied distal tendons of the EDL, the distal tendon of the TA, and the distal tendon of the EHL muscles were sutured to four separate Kevlar threads in order to provide connection to force transducers.

#### Experimental Set-Up

**FIGURE 1:**
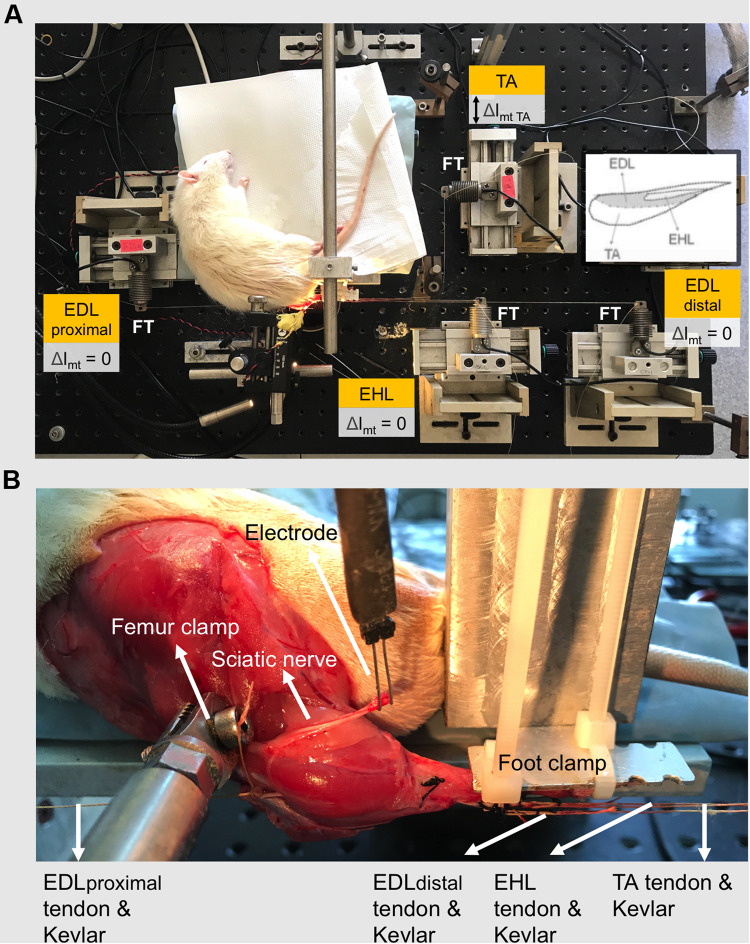
The experimental set-up. **(A)** The distal tendons of the TA and the EHL muscles as well as the proximal and the tied distal tendons of the EDL muscle (EDL proximal and EDL distal, respectively) were each connected to a separate force transducer by Kevlar threads. Throughout the experiment, the EDL and EHL muscles were kept at constant muscle-tendon complex lengths. Exclusively, the TA muscle was lengthened (Δl_mt TA_) to progressively increasing lengths, at which isometric contractions were performed. Lengthening (indicated by double-headed arrow) started from muscle active slack length at 1-mm increments by changing the position of the TA force transducer. Inset shows relative sizes and positions of muscles of the anterior crural compartment. **(B)** Experimental reference condition for joint angles are 120° and 100° for knee and ankle angles, respectively. The femur and the foot were fixed by metal clamps. The distal end of the sciatic nerve was placed on a bipolar silver electrode.

To mount the animal in the experimental set-up (Figure 1A) the following procedure was utilized: (1) in order to avoid obstruction of the Kevlar threads connecting the distal tendons to their force transducers, the ankle was brought to maximal plantar flexion (180°) in which position, the foot was fixed to the foot clamp. (2) The femur was fixed to the femur clamp such that the knee angle was set at 120°. (3) Taking care to ensure their alignments in the muscle’s line of pull, each Kevlar thread was connected to a separate force transducer (BLH Electronics Inc., Canton, MA, United States). (4) The distal end of the sciatic nerve was placed on a bipolar silver electrode (Figure 1B).

#### Experimental Conditions and Procedure

For the duration of the experiment room temperature was kept at 26°C. To prevent dehydration, muscles and tendons were irrigated regularly by isotonic saline. The distal and proximal tendons of the EDL and the distal tendon of the EHL muscles were kept in their reference positions at all times. Therefore, during the experiment, their lengths were not changed. However, the TA was brought to various muscle-tendon complex lengths, by repositioning its force transducer. The isometric forces of all muscles were measured simultaneously at each TA length. The measurement started at active slack length of the TA and its length was increased in 1 mm increments until reaching 2 mm over its optimum length. TA muscle-tendon complex lengths are expressed as deviation from its active slack length (Δl_mt TA_).

Subsequent to bringing the TA to a target muscle length, all muscles studied were activated maximally using a constant current of 2 mA (square pulse width 0.1 ms) delivered to the sciatic nerve (STMISOC; BIOPAC Systems, Goleta, CA, United States) with the following stimulation protocol: (1) two twitches were evoked. (2) 300 ms after the second twitch, the muscles were tetanized (pulse train 400 ms, frequency 100 Hz). (3) 200 ms after the tetanic contraction, another twitch was evoked. Each completion of this protocol was followed by a recovery period of 2 min for all muscles. During the recovery period, the TA was kept near the active slack length. However, the EDL and EHL lengths were not altered.

### Assessment of Changes in Intramuscular Connective Tissue Content Due to BTX-A

In a separate set of male Wistar rats, changes in intramuscular connective tissue content due to BTX-A were assessed. The animals were divided into two groups: control (*n* = 6; mean ± SD: body mass 404.3 ± 31.0 g) and BTX-A (*n* = 6; body mass 413.3 ± 46.2 g).

Collagen amount of each muscle was quantified using a colorimetric analysis of hydroxyproline content (Carlson, 2014) 1-month after the injections. Subsequent to above described surgical procedures to expose the anterior crural muscles, biopsies were removed rapidly after euthanizing the animal. Purity of the muscle samples were provided by careful removing of all tendinous materials from the sample. Muscle biopsies were flash-frozen in liquid nitrogen and stored at −80°C before running the assay within 4 weeks after removal. In short, each muscle was weighed prior to undergoing hydrolyzation at 130°C for 12 h in 5 N HCl. Samples of the hydrolyzate were oxidized at room temperature with a chloramine-T solution for 25 min incubation. Subsequently, the impurities were extracted and discarded by toluene treatment. To convert the oxidation product to pyrrole, the remaining aqueous layer containing the hydroxyproline products was heated for 30 min in boiling water. The final pyrrole reaction product is then removed in a second toluene extraction, and the final solution was mixed with Ehrlich’s reagent for 30 min. Sample absorbances were read at 560 nm in triplicate using UV-Visible spectrophotometer (UV-1280; SHIMADZU, Kyoto, Japan).

### Data Processing

**FIGURE 2:**
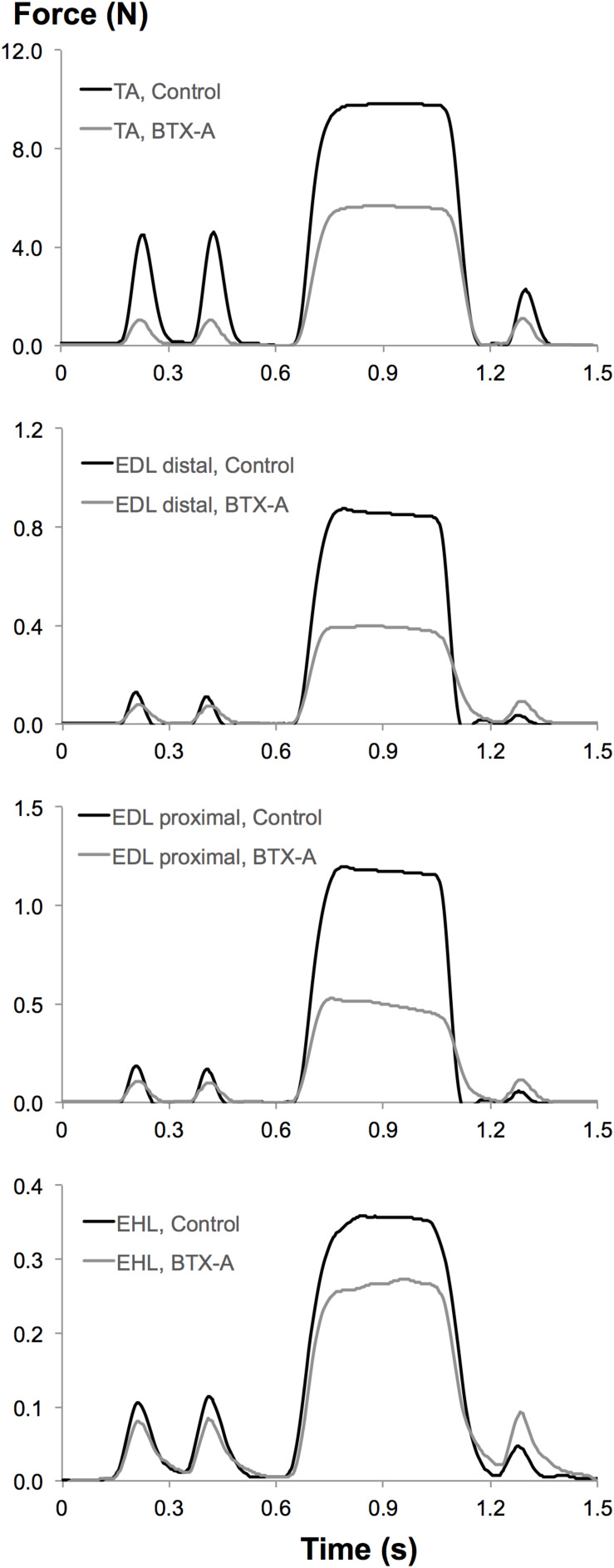
Typical examples of force-time traces measured at tendons of experimental muscles from both control and BTX-A groups. Upper to lower panels: superimposed force-time traces of the TA, EDL distal, EDL proximal, and EHL muscles recorded at the optimum length of TA muscle.

Muscular tissues’ capacity of mechanical resistance at a tested muscle-tendon length is characterized by muscle force in an unstimulated state. This is referred to as passive muscle force (*F*_*p*_). Using the force-time traces obtained experimentally: (i) *F*_*p*_ was determined 100 ms after the second twitch (Figure 2). (ii) Muscle total force was determined as the mean force (for a 200 ms interval, 150 ms after evoking tetanic stimulation) during the tetanic plateau. Muscle active force (*F*_*a*_) per muscle-tendon complex length was calculated by subtracting *F*_*p*_ from muscle total force.

A least squares criterion was used to fit the data for *F*_*p*_ and *F*_*a*_, with a polynomial function:

(1) $$y=a_{0}=a_{1}\,{\text{x}}^{2}+a_{2}\,{\text{x}}^{2}+\ldots +a_{\text{n}}\,{\text{x}}^{\text{n}}$$

where y represents isometric muscle forces (i.e., *F*_*p*_ or *F*_*a*_), and x represents muscle-tendon complex length. a_0_, a_1_…a_n_ are coefficients determined in the fitting process.

Using one-way analysis of variance (ANOVA), the order of the polynomials was determined (Neter et al., 1996). The lowest order was sought after with the criterion that a significant improvement was still provided to the description of changes in muscle force data as a function of muscle-tendon complex length. These polynomials were used for calculating mean and standard deviations (SD) of data: for each muscle studied, muscle forces at different TA muscle-tendon complex lengths were obtained. For each TA muscle-tendon complex length, muscle forces were averaged in order to determine the muscle force (mean ± SD) of the control and BTX-A groups.

Muscle length-force characteristics were studied using four key determinants defined as follows: (1) *Muscle optimal force* is the maximum isometric force exerted by an active muscle. Muscle optimal force is often taken as an indication of a muscle’s capacity for force production. (2) *Muscle optimum length* is the muscle length at which muscle optimal force is encountered. (3) *Muscle active slack length* is the shortest length at which an active muscle can still exert non-zero force. (4) *Muscle length range of force exertion* is the length range from active slack length to optimum length. Within the potential joint range of motion, this is considered as a metric indicating movement capability, with active force exertion.

Also to process these key determinants, the polynomials obtained were utilized in order to determine: the optimal TA force (i.e., the maximum active muscle force value of the fitted polynomial, for each individual TA muscle), the corresponding optimal muscle length, as well as TA active slack length. The TA length range of active force exertion (*l*_range_) was determined as the muscle length range between muscle active slack length and muscle optimum length.

Hydroxyproline analysis was used to quantify changes in intramuscular collagen content for muscles exposed to BTX-A. Using the measured absorbance values of muscle samples, hydroxyproline contents of individual muscles were determined based on a reference (i.e., standard regression curve identifying the paired information of pre-known hydroxyproline amounts and their measured absorbance values) as μg hydroxyproline expressed per mg of muscle tissue wet weight. Hydroxyproline content was converted into intramuscular collagen content using a constant (7.46), which characterizes the number of hydroxyproline residues in one molecule of collagen (Neuman and Logan, 1950).

### Statistical Analyses

After using Shapiro–Wilk test to seek for a normal distribution in *l*_range_ data of the TA, unpaired *t* or Mann-Whitney *U* test was used to test for the effects of BTX-A injection on this metric.

Two-way ANOVA for repeated measures (factors: TA muscle-tendon complex length and animal group) was performed separately for the forces of each muscle. If significant main effects were found, Bonferroni *post hoc* tests were performed to further locate significant within-factor differences.

Forces of both groups were aligned for their optimum length. Decrease in muscle active force is calculated per TA muscle-tendon complex length, as the difference in mean force between the control and the BTX-A groups. This is expressed as a percentage of the mean force of the control group. The Spearman’s rank correlation coefficient (ρ) was calculated to test if reductions in TA active forces due to BTX-A injection are correlated with TA muscle-tendon complex length. Correlations were considered significant at *P* < 0.05.

Shapiro–Wilk test was used to check if the collagen content data are normally distributed. The collagen amount calculated for each muscle in BTX-A group was compared to those of control group using unpaired *t* or Mann-Whitney *U* test, where appropriate. Differences were considered significant at *P* < 0.05.

## Results

### Effects of BTX-A on Muscular Mechanics

#### TA Force-Length Characteristics

**FIGURE 3:**
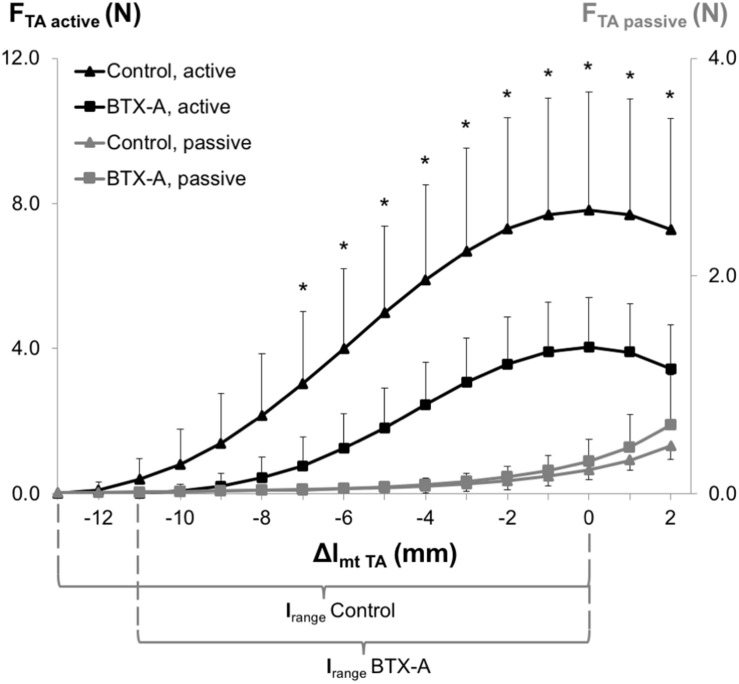
Forces of the TA as a function of increasing TA muscle-tendon length. Active and passive muscle forces are shown as mean and standard deviation values for the control and BTX-A groups. The TA muscle-tendon complex length is expressed as a deviation from its optimum length (Δl_mt TA_). Significant differences between the TA active force of the control group and BTX-A group (Bonferroni *post hoc* test) are indicated by *.

ANOVA (factors: TA length and animal group) showed both significant main effects on TA active forces, and a significant interaction. *Post hoc* testing showed significant effects of BTX-A for most muscle lengths (Δl_mt TA_ ≥ −7 mm). TA active force reductions (e.g., 75.2, 48.3, and 52.8%, respectively, at Δl_mt TA_ = −7 mm, Δl_mt TA_ = 0 mm and Δl_mt TA_ = 2 mm) were inversely correlated with increasing TA muscle length (ρ = −0.94, *P* < 0.001). The *l*_range_ of the BTX-A group (8.56 ± 2.05 mm) was significantly narrower compared to that of the control group (11.10 ± 1.58 mm) by 22.9%. ANOVA showed significant main effects also on TA passive forces, but no significant interaction. Compared to the control group, the passive forces are higher in the BTX-A group on average by 12.3% (Figure 3).

#### EDL Forces

**FIGURE 4:**
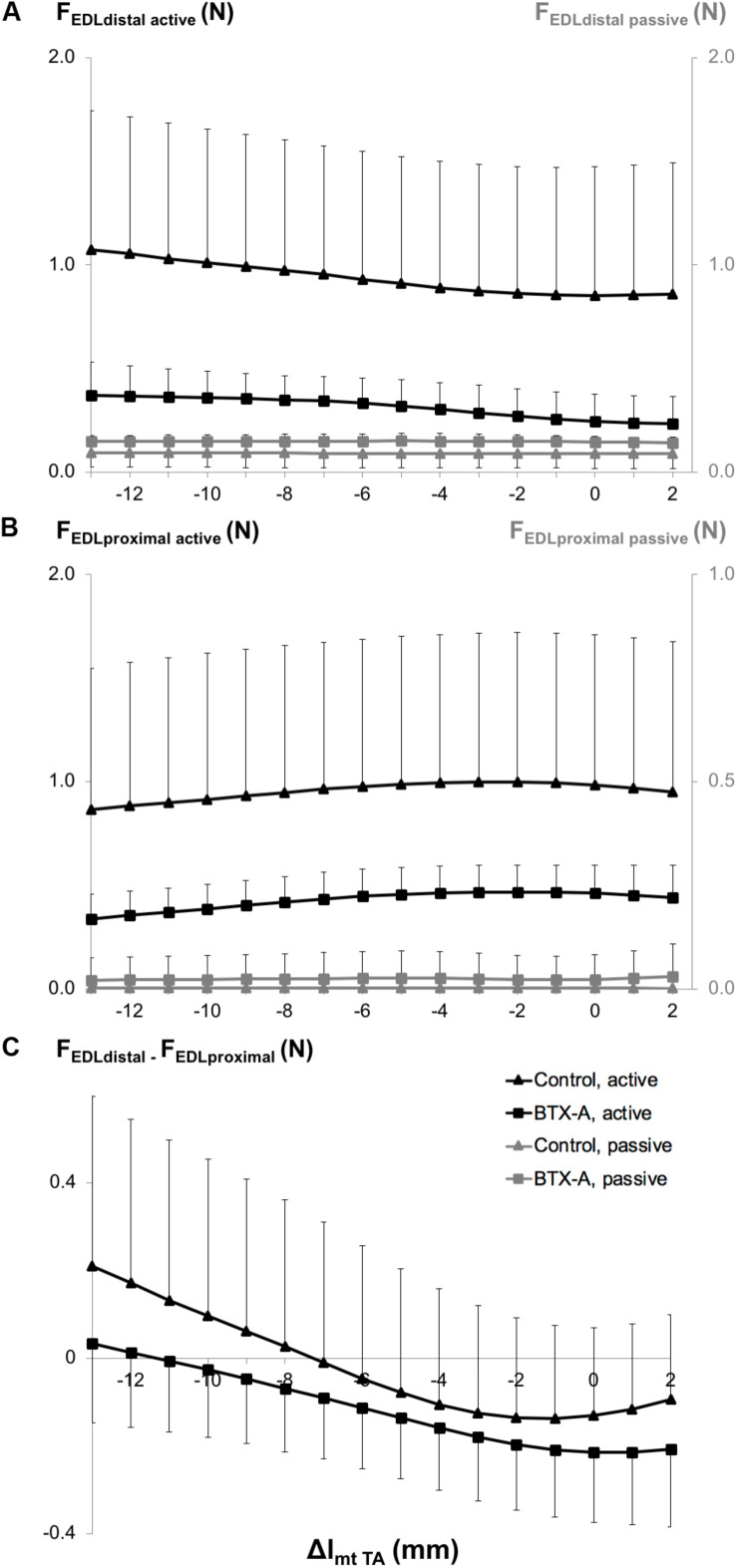
Forces of the EDL as a function of increasing TA muscle-tendon length. Active and passive forces of the EDL measured from **(A)** the distal tendon, and **(B)** from the proximal tendon, and **(C)** proximo-distal active force differences of EDL (*F*_EDL distal_ – *F*_EDL proximal_). Isometric muscle forces are shown as mean and standard deviation values for the control and BTX-A groups. TA muscle-tendon complex length is expressed as a deviation (Δl_mt TA_) from its optimum length.

ANOVA showed both distally and proximally, only a significant effect of animal group on EDL active and passive forces. However, neither significant effects of TA length nor a significant interaction were found. BTX-A caused significant active force decreases (on average by 66.8% distally and 55.4% proximally) and passive force increases (on average by 62.5% distally and more than twice proximally) compared to those of the control group (Figures 4A,B).

ANOVA also showed significant main effects of both factors on the EDL proximo-distal active force differences (Figure 4C), but no significant interaction. Whereas for the control group, both positive and negative proximo-distal active force differences were shown (distal EDL forces were higher than proximal ones for Δl_mt TA_ ≤ −7 mm and vice versa at longer TA lengths), for the BTX-A group, the EDL proximal forces were higher than the distal forces for most of the TA lengths (Δl_mt TA_ ≥ −11 mm). Bringing the TA to longer lengths changed the proximo-distal active force difference measured at the shortest muscle length (Δl_mt TA_ = −13 mm) significantly for Δl_mt TA_ ≥ −8 mm and for Δl_mt TA_ ≥ −9 mm, for the control and BTX-A groups, respectively.

#### EHL Forces

**FIGURE 5:**
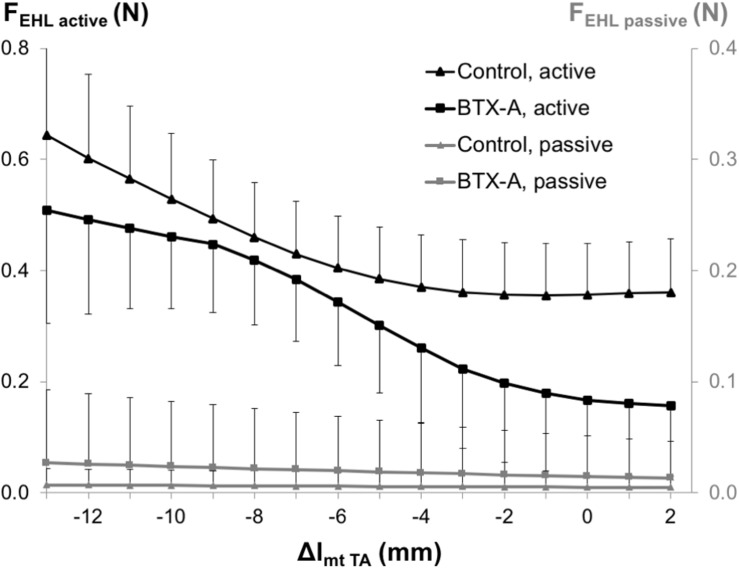
Forces of the EHL as a function of increasing TA muscle-tendon length. Active and passive muscle forces are shown as mean and standard deviation values for the control and BTX-A groups. TA muscle-tendon complex length is expressed as a deviation (Δl_mt TA_) from its optimum length.

ANOVA showed significant main effects of both factors on EHL active forces, but no significant interaction and only a significant effect of BTX-A on passive forces, but no significant interaction. BTX-A caused a significant active force decrease (on average by 28.8%) and passive force increase (on average by more than twice) compared to those of the control group (Figure 5).

### Changes in Intramuscular Connective Tissue Content Due to BTX-A

**FIGURE 6:**
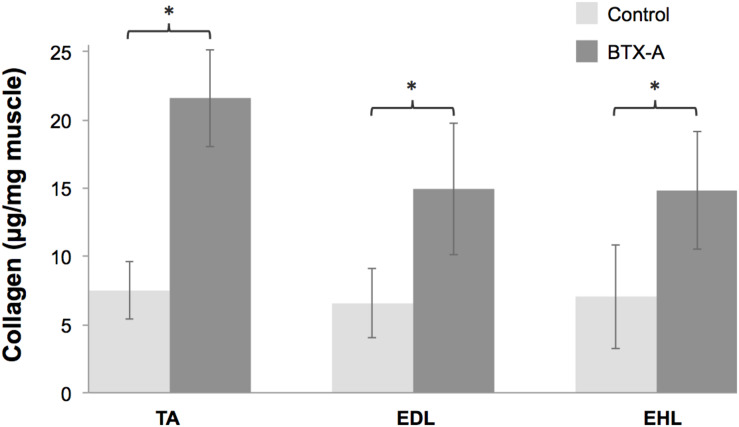
Collagen contents of the TA, EDL, and EHL muscles shown as mean and standard deviations for the control and BTX-A groups. Significant differences between the control and BTX-A groups are indicated by ^∗^.

For both injected TA muscle and non-injected EDL and EHL muscles, were the intramuscular connective tissue contents (Figure 6) significantly higher for the BTX-A group compared to those of the control group (BTX-A group: 21.61 ± 3.56, 14.94 ± 4.82, and 14.82 ± 4.32; Control group: 7.49 ± 2.08, 6.55 ± 2.55, and 7.05 ± 3.81 μg collagen/mg muscle; *P* = 0.005, 0.006, and 0.029 for the TA, EDL, and EHL, respectively). Note that, muscle masses in the BTX-A group were less than those of the control group (BTX-A group: 0.35 ± 0.04, 0.10 ± 0.01, and 0.01 ± 0.003 g; Control group: 0.65 ± 0.04, 0.16 ± 0.03, and 0.01 ± 0.01 g; *P* < 0.001, and *P* = 0.005, and 0.198 for the TA, EDL, and EHL, respectively).

## Discussion

Rejecting the first hypothesis, the present data indicate a reduced *l*_range_ for the injected TA muscle. The hypothesis was based on our previous short-term experiment (Yucesoy et al., 2012), which showed unlike an expected increase, no significant changes in the *l*_range_. However, this study showed that in the long-term, BTX-A leads to even a narrowing of the muscle’s *l*_range_. Elevated passive force of the injected TA muscle confirms the second hypothesis and is a consistent finding with the short-term effects of BTX-A shown earlier (Yucesoy et al., 2012). Active and passive forces of not only the injected muscle, but all muscles within the anterior crural compartment were altered indicating spread of BTX-A into also the non-injected muscles. This confirms the third hypothesis. Elevated intramuscular collagen combined with the muscle atrophy observed agrees with the increased muscle passive forces. Therefore, BTX-A induced changes to the structure and mechanics of both targeted and untargeted muscles persist and advance in the long term.

### Altered Mechanics of Muscles Exposed to BTX-A

**FIGURE 7:**
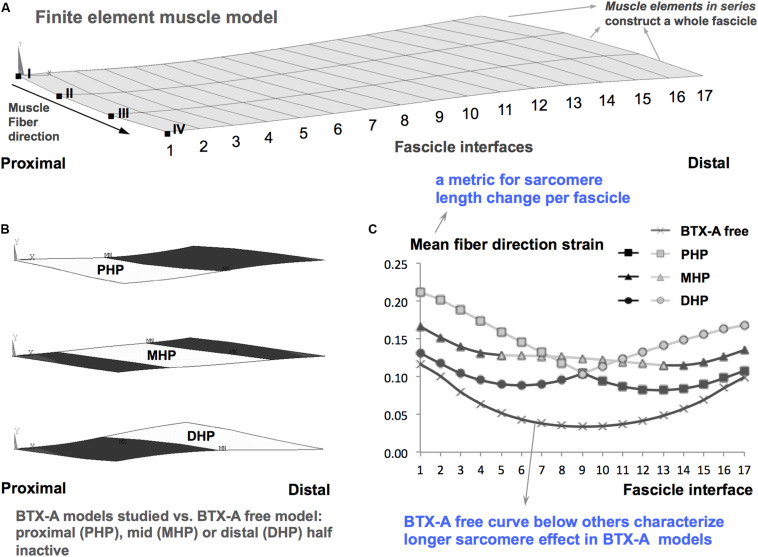
Longer sarcomere effect illustrated based on Turkoglu et al. (2014). **(A)** Finite element muscle model consists of combinations of muscle elements arranged in series each of which constructs a whole fascicle. A combination of model nodes along one side of a fascicle is referred to as a fascicle interface. For example, the most proximal fascicle interface is a combination of the nodes indicated by Roman numerals from I to IV. Each fascicle interface is indicated by a number from 1 to 17. The local length changes along the muscle fiber direction, i.e., fiber direction strain is analyzed. **(B)** Schematic representation of BTX-A models studied. These are achieved by not activating muscle elements (representing BTX-A induced partial paralysis) located in the proximal half (PHP), middle half (MHP), and distal half (DHP) of the muscle: white areas show paralyzed muscle parts, whereas the darker areas show the parts that are activated maximally. These models are studied versus a BTX-A free model (i.e., the entire muscle is activated maximally). **(C)** The characteristic BTX-A effect, i.e., the longer sarcomere effect shown earlier (Turkoglu et al., 2014), exemplified. Mean fiber direction strain is the average of local length changes in nodes I to IV in each fascicle interface. Therefore, it is a metric characterizing sarcomere length change per fascicle. Mean fiber direction strain curve for the BTX-A free muscle is localized below those of the BTX-A models indicating that sarcomeres within muscle exposed to BTX-A attain longer lengths.

The mechanism of effects of BTX-A on muscular mechanics and in particular how this reflects on muscle’s *l*_range_ has been explored using finite element modeling (Turkoglu et al., 2014). The characteristic determinant for this mechanism was indicated as the “longer sarcomere effect” (LSE). In short, the inactivated muscle fibers modeled, which represent BTX-A induced partial muscle paralysis does not shorten. This effect is reflected also to the activated ones via muscle fiber-extracellular matrix (ECM) mechanical interactions (Yucesoy, 2010; Yucesoy and Huijing, 2012) yielding overall a limited shortening of sarcomeres in muscle exposed to BTX-A compared to their counterparts in a BTX-A free muscle. Due to such LSE (please see Figure 7 for an illustration), the sarcomeres reach their maximal force production earlier causing the muscle’s optimum length to shift to a shorter length. This explains the narrowing of *l*_range_. Finite element modeling of the time course of BTX-A treatment further predicted that such effect of BTX-A may get more pronounced in the long-term (Turkoglu and Yucesoy, 2016). This was characterized by an increased stiffness of the muscle’s ECM, which, via a more pronounced LSE, was shown to cause sustained and/or increased shifting of muscle optimum length to shorter lengths. This mechanism is likely to explain the present experimental findings, which in contrast to no significant effects reported acutely (Yucesoy et al., 2012) did show a narrowed *l*_range_ for the TA exposed long-term to BTX-A. Based on those earlier studies we consider that operation of the sarcomeres at longer lengths within the BTX-A exposed muscle could plausibly be a mechanism explaining the present findings. However, specific new tests should be conducted in order to confirm that. Note that, significantly higher total collagen content of muscles exposed to BTX-A is an important present finding. This is in concert with the elevated muscle passive forces shown and also with the increased ECM stiffness considered in previous finite element modeling. The hydroxyproline analysis conducted objectively addresses changes in intramuscular connective tissue content indicating increased collagen in muscle exposed to BTX-A. As collagen is the main load-bearing constituent of the ECM, this analysis was directly complementary for our aim of assessing the effects of BTX-A on muscular mechanics. Yet, an assessment of the expression and orientation of specific collagen isoforms and other elastic proteins such as titin (Thacker et al., 2012) in future studies can make the analysis of BTX-A-induced structural and mechanical changes comprehensive.

### BTX-A Effects at Large and on Mechanical Interactions Between Muscles

BTX-A can affect intermuscular mechanical interactions in two ways: (1) As a highly diffusive toxin, BTX-A can spread beyond the injected muscle through muscle fascia (Shaari et al., 1991), blood stream (Ansved et al., 1997), and/or axonal pathways (Antonucci et al., 2008). Mechanical effects of leakage of BTX-A into a neighboring muscle (Yaraskavitch et al., 2008), into synergistic muscles within a compartment (Yucesoy et al., 2012, 2015; Ates and Yucesoy, 2014; Yucesoy and Ates, 2018), and even across antagonistic compartments (Ates and Yucesoy, 2018) have been reported in several animal studies. This resulted in at the least a dropped active muscle force, but also lead to elevated collagen content, stiffness, and passive force as well as a reduced *l*_range_ of non-injected muscles exposed acutely to BTX-A by diffusion. The effects of BTX-A investigated presently in the long term confirmed that BTX-A leads to a decrease in the active forces of all synergistic muscles within the compartment. On the other hand, BTX-A administration is clinically expected to improve agonist-antagonist force balance (Gracies et al., 2007; Elvrum et al., 2012). However, assuming that inter-compartmental spread of BTX-A occurs in patients and is effective in the long term, BTX-A effects on such imbalance may not be as simple as weakening of the target muscle for a better match with the force of the antagonist. Instead, mechanically this may involve also weakening of the antagonist. However, BTX-A may decrease cocontraction of antagonistic muscles, which is exaggerated in children with CP (Ikeda et al., 1998). Nevertheless, this may not be a controlled effect. More importantly, although positive effects of BTX-A against the neurological pathology are plausible, the present findings suggest that simultaneously occurring mechanical effects may not be favorable ones. (2) Muscles are connected to the joint through the tendons; however, their bellies are interconnected via an integral system myofascial connective tissue structures (e.g., collagenous connections providing integrity between the epimysia of adjacent muscles and collagen-reinforced neurovascular tracts interconnecting bellies of intra- and intercompartmental muscles). In BTX-A free conditions, epimuscular myofascial force transmission (EMFT) (Huijing, 2009; Yucesoy, 2010), i.e., intermuscular mechanical interactions occurring through those connective tissue structures impose major effects on muscular mechanics including the following: magnetic resonance imaging analyses indicate heterogeneous local length changes (varying, e.g., from 29% lengthening to 13% shortening) along muscle fibers of human medial gastrocnemius after imposing passive stretch (Pamuk et al., 2016) or submaximal activation (Karakuzu et al., 2017). Supersonic shear imaging analyses show similarly local muscle stiffness changes (e.g., the shear modulus of the TA was higher in extended knee compared to flexed knee position on average by 27%, despite the fact that ankle angle was restrained) (Ates et al., 2018a). Muscle mechanics experiments indicate changes in muscle’s length-force characteristics in response to altered mechanical conditions within which the target muscle operates: (i) In animal experiments, those changes due to co-activation of synergistic muscles include elevated agonist muscle force amplitude (e.g., by 17%) and shift of muscle’s maximal force production to a different length (e.g., by several mm’s, yielding an increase in muscles length range of force production by 24%) (Yucesoy et al., 2003a; Yucesoy and Huijing, 2007). (ii) In intraoperative experiments in CP patients, co-activation of synergistic and antagonistic muscles was shown to yield similar effects altering force production of the target spastic muscle compared to that measured after it is activated alone (Ates et al., 2018b; Kaya et al., 2018, 2019, 2020). However, previously, EMFT has been shown to be affected by BTX-A exposure in the short-term (Yucesoy et al., 2015). One characteristic effect of EMFT is the proximo-distal force differences (i.e., unequal muscle forces exerted at both ends of a biarticular muscle, reaching up to 35%) (Huijing and Baan, 2001; Yucesoy et al., 2003a, b). This represents the net amount of epimuscular myofascial loads (i.e., forces arising from stretching of the intermuscular connections due to muscle relative position changes) acting on the muscle (Yucesoy, 2010). The present results showed that exposure to BTX-A does affect proximo-distal force differences for the EDL muscle. At the shortest TA lengths, for both animal groups, distally exerted EDL active forces were higher than those exerted proximally. This indicates that proximally directed epimuscular myofascial loads act on the EDL belly. After imposing TA lengthening, the direction of those loads changed to become distally directed for both groups, but the amplitude of the net amount of epimuscular myofascial loads was higher for the BTX-A group. Previously, BTX-A injected into the TA was shown to acutely remove EDL proximo-distal force differences for all TA lengths studied (Yucesoy et al., 2012). Moreover, in a specific test involving solely relative position changes of the EDL, which was kept at constant length showed minimized EDL proximo-distal force differences indicating diminished EMFT after exposure to BTX-A (Yucesoy et al., 2015). Although the latter test was not conducted presently, the findings (i) in contrast, do not show a diminished EMFT in the long term, but judging from the elevated distally directed myofascial loads, and (ii) indicate that intermuscular connective tissue linkages between the TA and the EDL should be stiffer. This issue is relevant because for patients with CP, recent intraoperative experiments show that inter-synergistic EMFT (Ates et al., 2018b; Kaya et al., 2018, 2019, 2020) and also the inter-antagonistic EMFT (Ates et al., 2014, 2018b; Kaya et al., 2019, 2020) cause a significant increase in the force of spastic gracilis and semitendinosus muscles (above 30%, up to 70%). This suggests that the spastic hamstrings capacity to affect the pathological condition in the joint is changed by EMFT. Therefore, it is important to assess in new clinical studies if exposure to BTX-A in the long-term sustains or even elevates the stiffness of connective tissue structures, which provide intermuscular mechanical connections.

### Limitations and Implications of the Study

The established time to assess the chosen long-term effects of BTX-A is based on concepts related to the process of exocytosis. This process allows the release of acetylcholine into the synaptic cleft at the neuromuscular junction, and any intervention targeting that including exposure to BTX-A yields presynaptic blocking in signal transmission, hence muscle paralysis (Blasi et al., 1993; Brin, 1997; Kareem, 2018). de Paiva et al. (1999) showed that 4-weeks after BTX-A injection, no exocytosis occurs at the parent nerve terminals. However, (i) this changes beyond 1-month duration (e.g., at day 63 the authors showed recovery in exocytosis in the parent terminals) and (ii) formation of a network of nerve-terminal sprouts takes place after BTX-A injection, showing an increase over time. Therefore, our choice of assessment of BTX-A effects 1-month post injection allows avoiding exocytosis in the parent terminals for a consistent testing and is in concert with earlier animal studies (Longino et al., 2005; Minamoto et al., 2007; Yaraskavitch et al., 2008; Thacker et al., 2012). However, earlier research such as de Paiva et al. (1999) suggests that due to a dynamic exocytosis process, involving varying influences of the parent terminals and nerve-terminal sprouts, also further longer-term BTX-A effects need to be studied for a comprehensive understanding. Nevertheless, our present study sheds light to such understanding and indicates presence of novel mechanical effects that may affect the function of treated muscle.

Note the differences between the injection protocol, BTX-A dosage and volume used in the present animal study and in common clinical practice. The injected TA muscle was located by the manual placement method using anatomical landmarks and palpation. However, electrical stimulation, electromyography or ultrasound-guided injection of BTX-A is suggested for clinical application in order to improve the accuracy and specificity of localization, especially for deep and/or very small muscles (Chin et al., 2005; Wissel et al., 2009; Heinen et al., 2010; Walter and Dressler, 2014). Nevertheless, Picelli et al. (2012) showed that such guidance does not provide a better outcome compared to the manual placement method for superficial muscles. Accordingly, we were confident in localizing the superficial TA muscle presently and also in standardizing the injection location and depth as bound by the protocol described in the section “Materials and Methods.” On the other hand, the optimal clinical injection dosage per targeted muscle depends on the muscle volume, the degree of spasticity, and the level of the muscle’s involvement in the patient’s pathological pattern of joint movement (Molenaers et al., 2010). Heinen et al. (2010) reported a safe total dose range for children with CP as 1 to 25 U/kg bodyweight. In lower extremity muscles, clinical BTX-A doses vary between 3 and 6 U/kg (Koman et al., 1993; Frasson et al., 2012). However, presently, the BTX-A quantity injected approximates only 0.32 U/kg. Therefore, a direct comparison suggests that the experimentally used quantity is much less than those typically used in clinical applications. Also the presently injected volume (approximately 64 μl/kg) is less than the clinically utilized volumes (2.5–8 ml/kg) in lower limb muscles (Koman et al., 2000, 1993; Frasson et al., 2012). However, the findings indicate substantial drops in the measured forces suggesting effectiveness and therefore, appropriateness of the present dose for the study purposes. Note also the considerable variability in the dose and volume injected across animal studies, conducted on different species. Reported per kg of the animal, those values include 8.3 U and 0.83 ml in the mouse (Carli et al., 2009), 1–10 U and 0.1 ml in the rabbit (Borodic et al., 1994), and 3.5 U and 0.23 ml in the cat (Yaraskavitch et al., 2008). Therefore, in terms of the dose and injected volume of BTX-A, a general limitation for animal studies is the difficulty in building an explicit relationship with the clinical practice. This applies to the present study as well. Yet, animal studies do reveal major new phenomena and the present study indicates previously unknown remarkable long-term effects of BTX-A on mechanics of muscles exposed. However, those effects should be tested in the clinics.

Being also related to the dose administered, the issue of injection site also requires attention. Contrary to the common clinical practice, which typically involves multiple injection sites (Brin, 1997; Graham et al., 2000), presently only the mid-target muscle belly was injected. Taking into account high diffusivity of BTX-A (Shaari et al., 1991), dividing the total dose per muscle between multiple injection sites, can be effective in preventing the spread of the toxin to other muscles (Graham et al., 2000; Molenaers et al., 2010). The present injection protocol involving a single site into which the entire dose was administered may plausibly have facilitated leakage of the toxin to the adjacent non-targeted muscles. Yet, also for protocols involving multiple injection sites, spread of BTX-A was shown to occur beyond the injection site (Shaari et al., 1991). This makes controlling of the effects of the treatment difficult and was considered as a side effect (Graham et al., 2000; Hyman et al., 2000). In contrast, Frasson et al. (2012) suggested that the spread of BTX-A they showed from foot flexors to even antagonists could positively contribute to improving gait in patients with CP. Overall, such spread, which as indicated in previous animal studies can take place inter-compartmentally (e.g., Ates and Yucesoy, 2018), implies at least uncontrolled effects occurring in non-targeted muscles. This needs to be specifically tested in the long term. However, taking into account BTX-A effects adversely affecting the mechanics of the muscles exposed as shown presently, controlling leakage of the toxin appears to be quite important for controlling the outcome of the treatment. Particularly, spread of BTX-A into a bi-articular muscle has effects on both proximal and distal joints and hence may even manipulate the movement in a non-targeted joint. Therefore, structural and functional effects of BTX-A on muscles exposed also through diffusion are worth testing in the clinics.

## Conclusion

In conclusion, the present findings show that previously reported acute BTX-A effects persist and advance in the long term. A narrower *l*_range_ and elevated passive resistance of the targeted muscle are unintended mechanical effects of BTX-A, whereas spread of BTX-A into other compartmental muscles indicates the presence of uncontrolled mechanical effects. These findings can be clinically relevant, but should be studied in patients.

## Data Availability Statement

The datasets generated for this study are available on request to the corresponding author.

## Ethics Statement

The animal study was reviewed and approved by Committee on the Ethics of Animal Experimentation at Boğaziçi University.

## Author Contributions

CY and CK contributed to the conception and design of the study and wrote the sections of the manuscript. CK, EY, and ZA-D contributed to the acquisition of experimental data. CK performed the statistical analysis and wrote the first draft of the manuscript. All authors contributed to interpretation of data for the work, manuscript revision, read, and approved the submitted version.

## Conflict of Interest

The authors declare that the research was conducted in the absence of any commercial or financial relationships that could be construed as a potential conflict of interest.
